# A prospective study to determine the cost of illness for oral cancer in India

**DOI:** 10.3332/ecancer.2021.1252

**Published:** 2021-06-17

**Authors:** Arjun Gurmeet Singh, Devendra Chaukar, Sudeep Gupta, C S Pramesh, Richard Sullivan, Pankaj Chaturvedi, Rajendra Badwe

**Affiliations:** 1 Tata Memorial Centre and HBNI, Mumbai 400012, India; 2 Institute of Cancer Policy, Guy’s Hospital, St Thomas Street, London SE1 9RT, UK

**Keywords:** oral cancer, India, low- and middle-income countries, costing, cost of illness

## Abstract

India accounts for almost a third of the global burden of oral cancer, a situation worsened by the inability to afford care. When available, aid is often insufficient, and costing is based on informal estimations. This study objectively determines direct healthcare costs of oral cancer in India. The study was performed from a healthcare provider’s perspective using a validated bottom-up method. Care pathways were determined by prospectively observing the natural management of 100 oral cancer patients treated between October 2019 and March 2020. Specific costing categories were built across services, and apportioned values for each interaction was averaged. Costs of treatment and service utilisation were obtained using probabilistic sensitivity analyses. The unit cost of treating advanced stages (United States Dollar (USD) 2,717) was found to be 42% greater than early stages (USD1,568). There was an 11% reduction in unit costs with increases in socioeconomic status. Medical equipment accounted for 97.8% of capital costs, with the highest contributor being imaging services. Variable costs for surgery in advanced stages were 1.4 times higher than early stages. Compared to surgery alone, the average cost of treatment increased by 44.6% with adjuvant therapy. These results show that over the next decade, India will incur an economic burden of USD 3 billion towards the direct healthcare of oral cancer. Early detection and prevention strategies leading to 20% reduction in advanced stage disease could save USD 30 million annually. These results are critical to deliver a disease-driven and objective reform for oral cancer care.

## Introduction

Cancer is the second leading cause of death globally, according to the World Health Organization [1]. In 2018 alone, it accounted for an estimated 9.6 million deaths, with approximately 70% occurring in low- and middle-income countries (LMICs) [1, 2]. The true burden might be much more than reported as cancer registries in LMICs are estimated to represent less than 10% of the actual population [3]. The International Agency for Research on Cancer has reported that less than 4% of populations across Africa, Asia and South and Central America have satisfactory data compared with 80% in North America [4]. Cancer of the oral cavity contributes significantly to this disease burden and is a serious challenge, more so for the LMICs, with India accounting for almost a third of the global incidence (India, Bhopal: Age Standardised Rate per 100,000 – 20.9) [5]. This rate has increased by 68% in the past two decades, making it the most common cancer among Indian males [6]. The various subsites usually affected include upper and lower lip, inner aspect (International Classification of Diseases (ICD) C00.3-4), tongue (ICD C02), gum (ICD C03.0-3.1), floor of mouth (ICD C04), hard palate (ICD C05) as well as others and unspecified parts of mouth (ICD C06) [7].

There have been considerable advances in the diagnosis and management of oral cancers in recent decades. However, these advances have contributed to increased costs of treatment, significantly impacting the cost of care for both providers and out of pocket expenditure for patients. Furthermore, in most LMICs, accessibility to health services is low, which coupled with poor health literacy results in a majority of cases presenting with advanced disease [8, 9]. Approximately 10% of the patients have progressive disease that makes them untreatable and can only be offered palliative care [8, 9]. Most of those who receive some form of curative treatment are left unemployed due to the debilitating process of the disease and become an economic and psychological burden on their friends and family. Even patients with health insurance and/or government aid, conventionally seen as immune to the cost of care, face serious challenges as most schemes do not provide the actual amount needed for treatment. This eventually increases their out of pocket expenses, pushing a significant proportion of the patients themselves and their families into a never-ending cycle of debt.

To tackle this issue, the Indian government has increased budgetary allocations towards cancer-specific sectors. Publicly sponsored cashless health schemes such as Ayushman Bharat have been implemented at the national and state level [10]. Most insurance schemes in India base their costing on expert opinions and not on formal quantitative analysis. In fact, there are only a handful of cost of illness studies for cancer in LMIC settings [11–13]. A cost of illness analysis would provide invaluable data that can inform objective policy frameworks for the appropriate allocation of resources [14–16]. Hence, we sought to determine the direct healthcare costs of oral cancer at a single major tertiary provider in India and to subsequently build a reproducible costing model that can be used for other cancers and in other countries.

## Methods

### Study setting

This study was performed from a health care provider’s perspective to determine the direct healthcare costs in treating oral cancer using a validated method [17, 18]. It was performed in a high-volume tertiary care cancer public hospital representative of many similar hospitals across India. The research protocol was approved by the institutional review board and ethics committee of Tata Memorial Centre. Since this was an observational study with no planned intervention that would impact immediate patient care, a waiver of consent was obtained from the ethics committee after full review of the protocol. The subjects were treated between October 2019 and March 2020. Patients were managed as per the consensus guidelines of the National Cancer Grid, India [19]. Approval for the study was obtained from the Institutional Ethics Committee before enrolling patients with recently diagnosed, treatment naïve, operable oral squamous cell carcinoma between the age of 18 and 65 years. Patients with distant metastasis at presentation or those requiring non-surgical management were excluded from the study. The demographic details of the patients and their cancers were collected. The socioeconomic status according to the Modified Kuppuswamy scale and their mode of payment for cancer care was recorded [20]. Complications during treatment and stay in the hospital were also documented using the Clavien–Dindo scoring system [21]. Based on Yamane’s formula (*n* = *N*/1 + *N*(e)^2^; where *n* = sample size; *N* = population (119,992 oral cancers in India in 2018); e = 1–precision; precision = ±10%), the sample size was estimated to be 100 patients.

### Framework analysis and health systems data sources

**Supplementary Figure 1.**
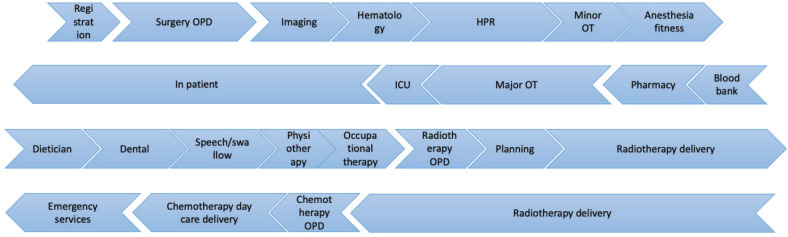
Oral cancer treatment framework.

The cancer care pathway was determined by prospectively observing sequential patients along their natural management pathway until the completion of cancer directed treatment, documenting each interaction with the relevant service (Supplementary Figure 1). Patients were stratified according to their stage at diagnosis into early (Stage I, II) and advanced (Stage III, IV) stage disease as per the latest American Joint Commission on Cancer (AJCC) classification. The pathway began with registration followed by consultation in the specified outpatient department with the pertinent diagnostic tests. Once the pre-treatment workup was completed, the patient was planned for appropriate surgical resection, with or without the need for appropriate reconstructive surgery. Their rehabilitation was augmented by supplemental therapies in the speech and swallowing department, occupational and physiotherapy and visits to the psychologist. Based on the postoperative histopathology report and staging of the disease, either observation, adjuvant radiotherapy (RT) alone or concurrent chemo-radiotherapy (CCRT) was prescribed. All complications, stay in hospital and other direct healthcare costs were captured through to completion of treatment.

Once the framework was established, data was collected to build each cost centre under specific costing heads such as fixed costs that included capital (diagnostic, medical and surgical), personnel and variable costs. Indirect costs, such as expenditure borne by the patient and family that were not directly attributable to oral cancer care, as well as intangible losses were not analysed. A facility survey was undertaken to assess the capital infrastructure, e.g. furniture, equipment, electronics, air conditioners, etc. Procurement prices of the diagnostic, medical and surgical equipment was obtained from the central store records of the hospital for the most recent year of asset purchase. Where there was lack of information, market prices were adjusted using averages to reflect the hospital procurement cost. The average life of the equipment was obtained either from the product catalogue or information from the standards set by the Income Tax Authorities of India and user dependent experience [22]. We did not include the land and the building costs as these largely vary depending on the geographic location and would inaccurately contribute to the broad assessments. Data on personnel salaries including benefits were obtained from the hospital’s human resources and accounts departments. For every service rendered, time allocation data on the interacting personnel was obtained from observation-based data and semi-structured interviews on a routine or fixed basis. The overhead costs were further divided into electricity, water, kitchen and laundry. Transportation costs were not included as they did not contribute towards the direct costs during the framework observations. The electricity consumption (kW/hour) utilised by each asset was obtained from the respective product catalogues and based on actual measurement of load in each service. The water consumption (litres) was calculated by the apportioned utilisation of each asset and the average consumption by various individuals activities in accordance with the Central Ground Water Authority of India [23]. The average cost of a meal was obtained from the Department of Dieticians. The cost of laundry was apportioned using the number of pieces laundered for each service. Variable resources in the form of non-medical and medical consumables and medications were documented at each interaction with the respective service and their retail price was obtained from stock registers, vouchers and pharmacy records of the hospital.

### Data analysis

The capital expenditure was annualised taking into account the asset depreciation allowance as per the Income Tax Department of India (Section 32, Income Tax Act) and the lifespan of the equipment [22]. Since some personnel could be jointly involved in overlapping activities, cost allocation for a particular service was based on observations and interviews to determine time contributions. The respective gross salaries were apportioned to each interaction with an assumption of an 8-hour work day for 6 days a week, equating to 229 days a year. Similarly, both capital and certain variable resources were apportioned using appropriate statistics. For overhead costs, the apportioned units were charged according to the actual rates and billing structures levied by the local authorities [24, 25]. The apportioned values for each interaction were added together and an average cost per service along the care pathway was obtained.

All the figures in the results section have been calculated in Indian National Rupees (INR) for the year 2018–2019 and were converted to USD based on the conversion rate of USD1 = INR74.68, according to the World Bank as of 18 August 2020 [26]. All figures are rounded up to the nearest whole number and hence might not always add up to a 100%. To factor in the uncertainty and variability of the estimates, we performed a multivariate probabilistic sensitivity analysis in which the costs were varied using conventional distributions. From the above estimates, an average unit cost of oral cancer along with unit service utilisation cost with a 95% confidence interval was calculated.

## Results

### Patient demographics

One hundred patients with a median age of 47 years (Range = 24 to 65 years), with equal numbers of early (48% Stage I, 52% Stage II) and late stage (34% Stage III, 66% Stage IV) were recruited between October 2019 and March 2020. The majority were males and only 13% (*n* = 13) of the cohort were female. The majority of patients, both early (70%) and advanced (86%) stage were from a middle-class status, with 53% depending on government insurance schemes to avail treatment. The location of oral cancers included tongue (*n* = 43), buccal mucosa (*n* = 35) and lower alveolus (*n* = 15). Most (63%) underwent some form of reconstruction that included local (28.6%, *n* = 18), regional (41.2%, *n* = 26) and free tissue transfer (30.2%, *n* = 19). The highest rate of Clavien–Dindo grade III/IV complications was seen among the regional flaps (15.4%, *n* = 4/26) followed by the free tissue transfer group (10.5%, *n* = 2/19) and local flap reconstructions (3.6%, *n* = 2/55). Although not statistically significant, there was a three-fold higher incidence of Clavien–Dindo grade III/IV complications in the advanced stage cancers than the early stages (12% versus 4%, *p*-value = 0.269). The median hospital stay for early and advanced stage disease was 7 and 8 days, respectively (Range = 3 to 22 days). Adjuvant therapy was prescribed for 51% of patients, with RT alone in 35% and CCRT in 16%. Six of the early stage patients (11.7%) received adjuvant therapy for adverse pathologic features not included in the AJCC staging system; that is for positive or close margins, extensive perineural invasion or poor grade of differentiation. Three patients with advanced stage disease (5.8%) could not complete their prescribed adjuvant therapy due to treatment delays.

### Per unit costs

**Figure 1.**
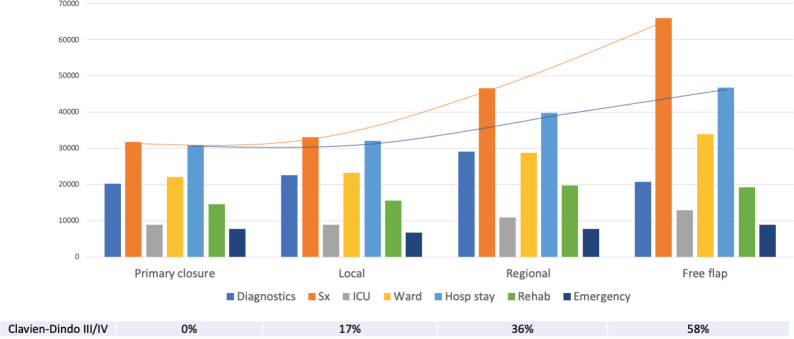
Cost distribution based on the type of reconstructive surgery performed and the complication rate.

**Figure 2.**
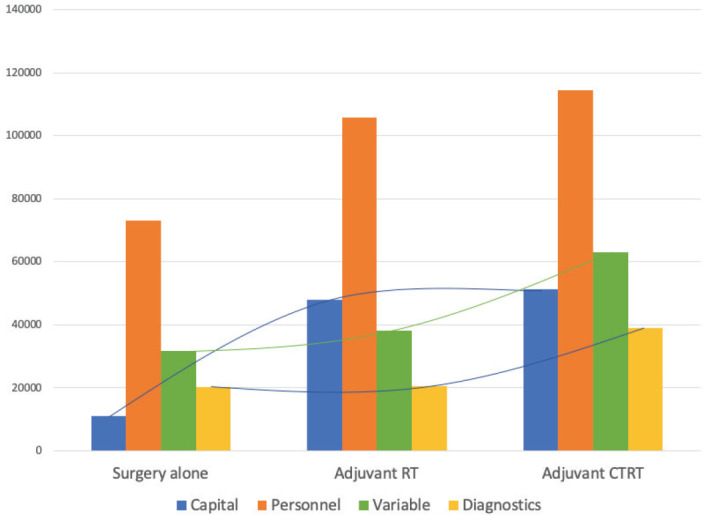
Cost distribution based on the modality of treatment received and the costing categories.

A total of INR16,001,368 (USD214,237) was the direct healthcare costs to treat 100 patients with oral cancer. Of this, the salaries of healthcare personnel contributed to 56.9% of the total costs followed by the variable (24.2%) and capital costs (18.9%). The personnel costs for the operating theatre (27.1%) were the highest followed by the in-patient department (24%). The highest variable costs were during the operation theatre utilisation, especially for long surgeries involving major reconstruction. The majority of the capital cost were medical equipment related (97.8%), with the highest proportion of per unit usage cost being contributed from the Magnetic Resonance Imagining (MRI) services. The unit cost of treating early and advanced stage disease was INR117,135 (USD1,568; Range = USD1,072 to USD2,620) and INR202,892 (USD2,717; Range = USD1,409 to USD3,813), respectively. The cost per unit based on stage of disease and the respective costing category is shown in Table 1. Adjusting for the six early stage patients that received adjuvant therapy and the three advanced stage patients that did not receive adjuvant therapy, the unit cost of early and advanced stage disease was INR108,715 (USD1,456; Range = USD1,072 to USD2,337) and INR202,603 (USD2,713; Range = USD1,409 to USD3,813), respectively. A 17% average increase in total unit cost was seen as the disease progressed through each stage from I to IVB, with the highest increment of 28% from stage II to III. At the same time, there was an average reduction of 11% in the unit costs as socioeconomic status increased. This could be due to the higher proportion of advanced stage cancers in the low and middle class, resulting from the significantly higher number of lesser educated individuals (completed high school, middle school, primary school and illiterate) in the group (*p*-value = 0.04). Since no upper class individuals availed any schemes, an 11% increase in the unit cost was seen when the patients were reimbursed (*p*-value = 0.000). The costs associated with the different types of reconstruction are shown in Figure 1. These costs were least when no major reconstruction was performed and highest for free tissue transfer. The cost of inpatient stay increased proportional to the rate of Clavien–Dindo III/IV complications. Compared to surgery alone, the average cost of treatment increased by 39.7% with adjuvant RT and by 49.5% when adjuvant CCRT was prescribed. The staff costs increased proportional to the addition of modalities of adjuvant therapy. The capital expenditure increased considerably with the addition of RT, but plateaued with the addition of chemotherapy (CT). Diagnostics and variable costs significantly increased with the addition of CT (Figure 2).

### Service costs

**Figure 3.**
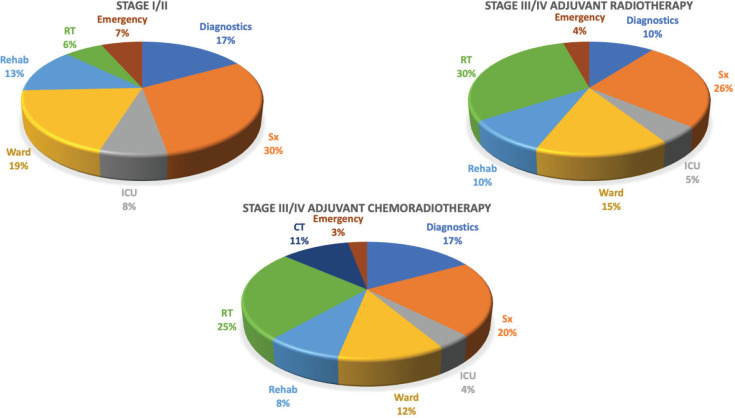
Cost distribution based on the services utilised.

Average costs for each service is described in Table 2. In early stage disease, surgery constituted 30% (INR35,436, USD475; Range = USD304 to USD1,085) of the overall costs followed by the in-patient department (19%, INR22,823, USD306; Range =USD114 to USD451) and diagnostics (17%, INR19,827, USD265; Range = USD158 to USD373). For advanced stage disease where only RT was prescribed, the adjuvant therapy constituted almost a third (30%, INR58,302, USD781; Range = USD689 to USD967) of the costs followed by surgery (26%, INR49,727, USD666; Range = USD323 to USD1,265) (Figure 3). When CT was added to the prescribed therapy, the adjuvant treatment costs increased by 6% with CT delivery alone constituting 11% (INR24,280, USD325; Range = USD75 to USD526) of the total costs. The highest average capital cost across all services was for RT among early and advanced stage (INR4,342, USD68 for early stage and INR32,007, USD429 for advanced stage, respectively) followed by imaging studies (INR3,875, USD52 and INR5,404, USD72, respectively). The highest average staff salaries (INR20,822, USD278 for early and INR28,431, USD381 for advanced stage) were incurred for surgery. The variable costs for surgery were 1.4 times and imaging studies were 1.6 times higher for advanced stage than early stage disease.

## Discussion

Oral cancer imposes a significant fiscal burden on a national, institutional and individual level. In 2020, India had an estimated incidence of 135,929 oral cavity cancers in 2020 that is expected to increase by around 26% in 2030 [5]. Moreover, 60%–80% of the cases present at advanced stages of the disease [8]. Multiplying the median cost per unit of early and advanced cancer as per our results, India spent approximately USD 322 million in 2020 on oral cancer treatment, paid for by insurance schemes, government and private sector spending, out of pocket payments and charitable donations or a combination of these. Without mitigation and assuming no appreciation in costs, this will mean an economic burden on the country of USD 3.2 billion over the next 10 years. Considering the major impact this will have on society, there is an urgency to identify appropriate pathways and efficiencies for disease management of oral cancers, including health technologies assessment(s). This study differs from the few previous economic assessments in that the estimates are prospectively based and not on those generated by decision-analysis models that often involve numerous assumptions. The accuracy of this costing method was further heightened by utilising a bottom-up approach where data was collected prospectively for each service as it was used, as opposed to the top-down approach where the total amount spent is usually apportioned based on the number of users in retrospect.

Successful treatment of patients with oral cancer is predicated on multidisciplinary treatment strategies to maximise oncological control and minimise the impact of therapy on form and function. One of the possible reasons for varied treatment costs in LMICs is the wide range of management protocols offered [27]. To mitigate this disparity, a care pathway was established for each stage based on widely accepted management guidelines. This is the first costing analysis based on individualised care pathways based on consensus guidelines of the National Cancer Grid that reflects best practices globally [19]. The cost of managing advanced stage cancer was found to be 42% greater than early stage tumours, with a 17% average increase in total unit cost as the stage progressed. These trends are similar to few other studies conducted around the world, showing that late stage disease disproportionally impacts budgets [28–31]. The need for composite surgery and adjuvant therapy increases proportionate to the stage of disease. Multimodality treatments that include RT and complex reconstructions have been shown to negatively impact the quality of life [32, 33]. Our work reinforces the importance of developing care pathways and management guidelines that are fit-for-purpose in resource constrained settings [34]. It is important to note that these costs were arrived at without including the land and building costs that are quite varied based on the location.

The economic impact of oral cancer treatment for India, and globally, strongly suggests that prevention must be one of the key mitigation strategies for addressing affordability. Oral cancers are among the diseases that are routinely amenable for simple visual screening measures [35]. Based on the results of a large randomised trial determining the effect of screening on oral cancer mortality, targeted screening may not only be cost effective, but also adds a significant preservation of quality adjusted life [36]. An institution audit has also reported a significant difference in 5-year survival outcomes between early and advanced oral cancer [37]. Patients in our study with advanced cancers were younger than early stage cancers by a median of 6 years. Hence, if these cancers were diagnosed and treated earlier, the median age would have shifted to the older age group leading to a substantial reduction in losses to the economy, as well as less spend on direct healthcare costs. Based on our results, by effective early detection and reducing the number of patients presenting in advanced stages by 20%, the direct healthcare costs to the country could be reduced by approximately USD 31 million annually. Reforms also need to target the most common aetiology behind a majority of oral cancers, i.e. smoked and smokeless tobacco, areca nut and alcohol use [38].

Our findings around the treatment costs of oral cancer need consideration in the wider architecture of cancer care in India. Around 40% of the comprehensive cancer centres are situated in the top eight metro cities and a majority (about 85%) are owned by trusts, private and corporate chains [39]. India has a very low oncologist to patient ratio (16 times lower in India than the United States), almost half of which are concentrated in the metropolitan areas [39]. This skewed geographic distribution limits access to advanced and multimodal treatment options making patients and families travel and lodge across the country, eventually increasing their out of pocket expense in addition to direct care costs [8].

Whilst our estimates are drawn from a single centre, they closely reflect economic reality in India. To date, most economic data feeding India’s insurance schemes has been drawn from reimbursement records. However, an estimate cannot be made by sifting through the state nodal records of the monetary sums handed out in the form of reimbursements, as most often this money only covers the cost of the limited enlistments. The cost of treating oral cancer in a large and populous country like India also needs to be seen in the wider context of healthcare costs in LMIC. The five Brazil, Russia, India, China, and South Africa countries comprise more than 40% of the world’s population and 25% of the global Gross Domestic Product (GDP) [26]. The economic losses due to cancer related mortality among just these countries were nearly 46.3 billion in 2012 [40]. Being one of the largest and fastest growing economies, a 1.29% GDP (as per Financial Year 2019–20) spending on healthcare services lands India at the tail end of the global list [26]. India recorded an astounding total productivity loss of 6.7 billion USD because of cancer alone, representing 0.36% of the GDP [40]. This loss was only second to South Africa that recorded 0.49% of its GDP lost to cancer. Interestingly, the productivity losses due to premature mortality and morbidity in LMICs have been shown to be the highest due to lip and oral cancer, with India leading at an astounding total of USD 740 million (USD30,563 loss/death) [40]. In fact, these costs are rapidly rising as younger individuals are being afflicted more commonly in India compared to western countries. Since most patients are at an economically productive age, this debilitation results in higher indirect losses due to premature disability and death. Using a human capital approach and the World Bank’s labour force statistics for India, the median age of diagnosis (47 years) and a reported 5-year survival rate of 50% would result in a productivity loss of approximately USD 310 million at the end of 5 years [26, 41, 42]. When all these costs are added together, the result is a catastrophic loss to the economy, let alone the intangible human loss of pain and suffering.

Direct treatment costs come hand in hand with out of pocket expenditures by patients and families in India. The approximate average spend on healthcare is USD85 per capita which is three to four times lower than neighbouring LMICs like Sri Lanka, China and Thailand and far from the High-Income Countries (HICs) [43]. With the per capita income at about USD1,600, the central and state government in India have made provisions to combat these cancers for people below a certain income level [26]. To add to this, there is a large disparity between the expenditure borne by an individual to avail cancer care in a private or public hospital. As per the Ministry of Statistics and Programme Implementation’s survey for the Key Indicators of Social Consumption in India, the average medical expenditure in a private hospital was a little more than 4 times that of a public hospital [44]. Our study resulted in an estimated cost of oral cancer treated at a semi aided facility, the true impact, combining private and public care, would be significantly higher than projected. Besides using their income and savings, people borrow money or sell their assets to meet their healthcare needs, eventually pushing 60 million people into poverty each year [45]. Public expenditure on cancer in India still remains below USD10 per person (compared with more than USD100/person in HICs), and overall public expenditure on health care is still under 1.5% of the GDP [34]. With the objective of health for all, the government has introduced novel schemes such as the ‘Ayushman Bharat’ [45, 46]. These insurances were conceived to reduce the financial burden on the poor and vulnerable groups arising out of catastrophic hospital expenses and ensure their access to quality health services. Under the world’s most anticipated and largest state-funded framework of healthcare, the government has capped procedural treatment costs for over a thousand empanelled cancer treatment packages, ranging from USD15 to over USD2,000 [45]. These rates are not based on objective evidence of the true cost of cancer management in the public system. One of the only other Indian studies on the cost analysis of different services involved in managing head and neck cancers found a cost of USD773 to perform a single head and neck surgery [47]. Based on a reasonable estimate of relevant packages for oral cancer management, the Pradhan Mantri Jan Arogya Yojana reimbursements would range from USD295 to USD1,018 for early oral cancer and USD1,808 for advanced cases. This is about 1.5 times lower than the estimates that have been derived from the current results highlighting a need for reconsideration in government pricing policy for oral cancer.

Although the intent to provide cancer treatment has been an important public health agenda item, the commitment cannot proceed very far without understanding the socioeconomic patterns, treatment seeking and financing behaviours. The limitations of this study include a possibility of under and/or over estimations of certain cost items, it was performed in a semi aided facility assumed to be representative of the wider population and that other cost objects, such as indirect losses, were not calculated to provide a true cost of illness estimate.

## Conclusion

Our cost estimates provide in-depth information that can assist policy makers in planning treatment disbursements more effectively. In a country where the affordability gap is wide, these results are important to deliver a disease-driven, objective strategy for oral cancer care. Moreover, the care pathways and costing model developed in this study can add to the evidence available and serve as a template for other cancers in various settings across countries.

## Conflicts of interest

We do not have any financial and personal relationships with other people or organisations that could inappropriately influence their work. There are no conflicts of interest to declare.

## Authors’ contributions

All authors have contributed equally.

## Funding statement

No funding was obtained for this work.

## Figures and Tables

**Table 1.** Cost distribution based on the stage of disease and the costing headings.

	Capital INR (USD)	Personnel INR (USD)	Variable INR (USD)	Total INR ± 1SD (USD ± 1SD)
Stage I	10,427 (140)	66,576 (891)	28,670 (384)	10,5673 ± 22,232 (1,415 ± 298)
Stage II	19,983 (268)	77,910 (1,043)	30,556 (409)	128,448 ± 36,665 (1,720 ± 491)
Stage III	42,504 (569)	101,185 (1,355)	34,916 (467)	178,605 ± 33,066 (2,392 ± 443)
Stage IVA	45,595 (610)	11,3476 (1,519)	52,331 (701)	211,553 ± 34,343 (2,833±460)
Stage IVB	51,232 (686)	115,353 (1,545)	60,853 (815)	227,438 ± 27,857 (3,045±373)
All stages	33,948 (455)	94,900 (1,271)	41,465 (555)	170,343 ± 52,354 (2,281 ± 701)

SD, Standard deviation

All values are rounded and hence might not add up to 100%

**Table 2.** Average costs per service in the framework.

Service	Early stage I/II				Advanced stage III/IV			
	Capital INR (USD)	Personnel INR (USD)	Variable INR (USD)	Total INR (USD)	Capital INR (USD)	Personnel INR (USD)	Variable INR (USD)	Total INR (USD)
Surgery outpatient department	1,660 (22)	4,840 (65)	164 (2)	6,664 (89)	1,745 (23)	5,085 (68)	172 (2)	7,002 (94)
CT scan	1,473 (19)	344 (5)	726 (10)	2,543 (34)	2,978 (40)	696 (9)	1,468 (20)	5,142 (69)
MRI scan	2,203 (30)	261 (4)	442 (6)	2,906 (39)	2,203 (30)	261 (4)	442 (6)	2,906 (39)
Haematology	190 (3)	2,000 (27)	839 (11)	3,029 (41)	423 (6)	4,377 (59)	1,836 (25)	6,636 (89)
Histopathology	293 (4)	987 (13)	341 (5)	1,621 (22)	293 (4)	987 (13)	341 (5)	1,621 (22)
Minor theatre	198 (3)	301 (4)	256 (3)	755 (10)	231 (3)	351 (5)	298 (4)	881 (12)
Major theatre	1,384 (19)	20,822 (279)	13,231 (177)	35,436 (475)	1,889 (25)	28,431 (381)	19,045 (255)	49,365 (661)
Inpatient Ward	226 (3)	19,070 (255)	3,527 (47)	22,823 (306)	292 (4)	24,627 (330)	4,860 (65)	29,779 (399)
Intensive care unit	299 (4)	5,499 (74)	3,165 (42)	8,963 (120)	395 (5)	7,263 (97)	3,713 (50)	11,371 (152)
Dietician	13 (0)	1,099 (15)	2,210 (30)	3,322 (44)	23 (0)	1,895 (25)	3,811 (51)	5,728 (77)
Dentist	86 (1)	2,099 (28)	537 (7)	2,722 (36)	108 (2)	2,645 (35)	677 (9)	3,430 (46)
Speech and swallow	242 (3)	3,466 (46)	53 (1)	3,762 (50)	264 (3)	3,779 (51)	58 (1)	4,101 (55)
Physiotherapy	165 (2)	2,297 (31)	150 (2)	2,611 (35)	173 (2)	2,404 (32)	157 (2)	2,734 (37)
Occupational therapy	67 (1)	748 (10)	1,606 (22)	2,420 (32)	69 (1)	776 (10)	1,667 (22)	2,512 (34)
Psychology	22 (0)	271 (4)	0 (0)	293 (4)	24 (0)	294 (4)	0 (0)	318 (4)
RT OPD	26 (0)	674 (9)	6 (0)	705 (9)	184 (3)	4,845 (65)	40 (1)	5,069 (68)
RT delivery	4,310 (58)	1,754 (23)	64 (1)	6,127 (82)	31,793 (453)	12,937 (173)	470 (6)	45,200 (605)
CT OPD	0 (0)	0 (0)	0 (0)	0 (0)	21 (0)	698 (9)	10 (0)	729 (10)
CT daycare ward	0 (0)	0 (0)	0 (0)	0 (0)	42 (1)	426 (6)	6,087 (82)	6,555 (88)
Emergency department	1,925 (26)	4,615 (62)	1,166 (16)	7,706 (103)	1,929 (26)	4,626 (62)	1,169 (16)	7,723 (103)

All values are rounded and hence might not add up to 100%

## Supplementary material

**Supplementary Table 1. Overhead cost.**

Available at https://doi.org/10.6084/m9.figshare.14779692.v1.

**Supplementary Table 2. Capital cost.**

Available at https://doi.org/10.6084/m9.figshare.14779695.v1.

**Supplementary Table 3. Final tally book.**

Available at https://doi.org/10.6084/m9.figshare.14779698.v1.

**Supplementary Table 4. Variable.**

Available at https://doi.org/10.6084/m9.figshare.14779701.v1.

**Supplementary Table 5. Personnel.**

Available at https://doi.org/10.6084/m9.figshare.14779704.v1.
